# Acute Hemoptysis Redefined: A Deadly Presentation

**DOI:** 10.1155/2018/2123140

**Published:** 2018-09-24

**Authors:** Claudio Galvis, Juan M. Galvis, Juan Guardiola, Adrian P. Umpierrez De Reguero

**Affiliations:** ^1^Departamento de Medicina de Emergencias, Hospital Teodoro Maldonado Carbo, Guayaquil, Guayas, Ecuador; ^2^Department of Pulmonary, Critical Care, and Sleep Disorders Medicine, University of Louisville School of Medicine, Louisville, Kentucky, USA; ^3^Department of Medicine, Division of General Internal Medicine, Section of Hospital Medicine, Medical College of Wisconsin, Milwaukee, Wisconsin, USA

## Abstract

An aortic aneurysm is a permanent localized arterial dilation with more than 50% of the artery diameter. Among the complications of an aortic aneurysm, one of the rarest is the aorto-bronchial fistula, which presents with massive hemoptysis; this condition is lethal if not treated surgically. We report a 90-year-old man with no significant medical history who presented to the emergency department with abrupt onset of hemoptysis; his chest X-ray displayed left upper lobe opacity with widened mediastinum. CT chest revealed aneurysmatic dilatation of the aorta, left upper lobe opacity suspicious of pulmonary aortic fistula. Thoracic surgery was consulted but due to his poor functional status surgery was deferred. On the second day of hospitalization, the patient developed another episode of massive hemoptysis resulting in hypovolemic shock and expired. This case epitomizes the relevance of broad differential diagnosis for hemoptysis and the prompt assessment and management of the patients with this condition.

## 1. Case

**Figure 1:**
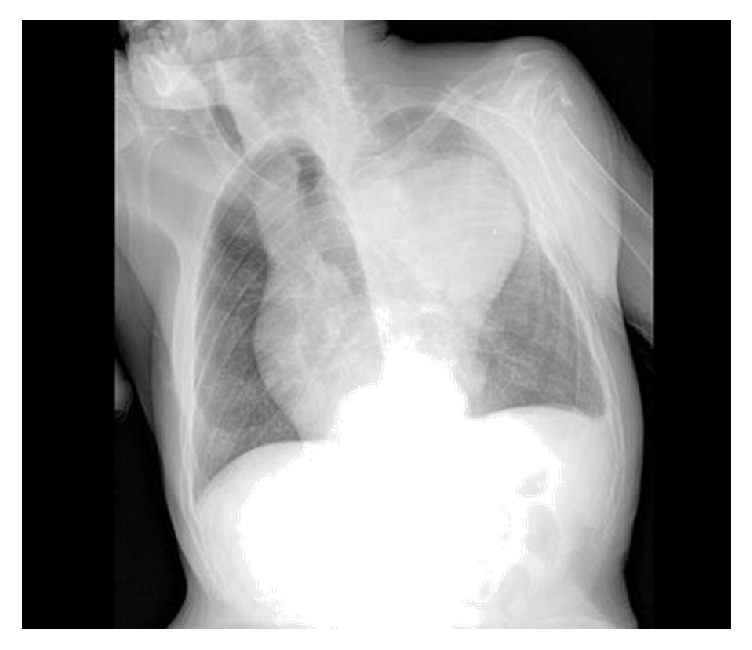
Mediastinal widening and left upper lobe opacity.

**Figure 2:**
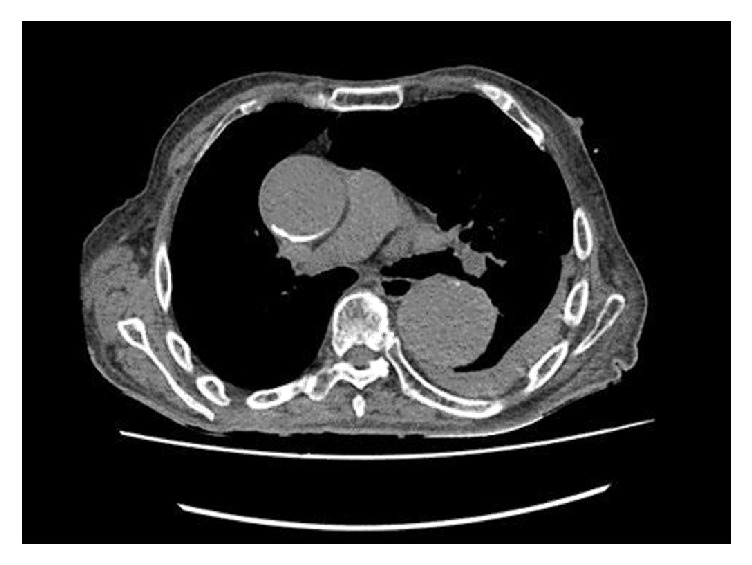
Aortic aneurysm.

**Figure 3:**
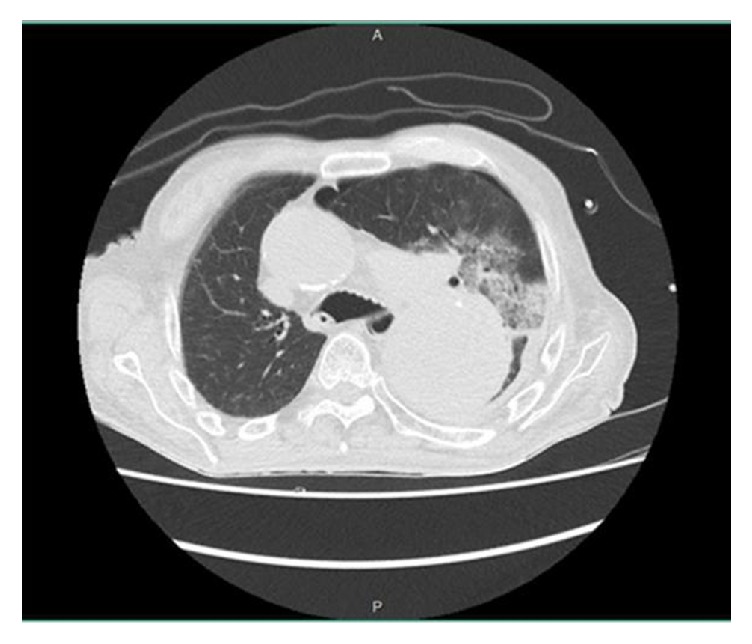
Aortic aneurysm with extravasation blood on lung parenchyma.

**Figure 4:**
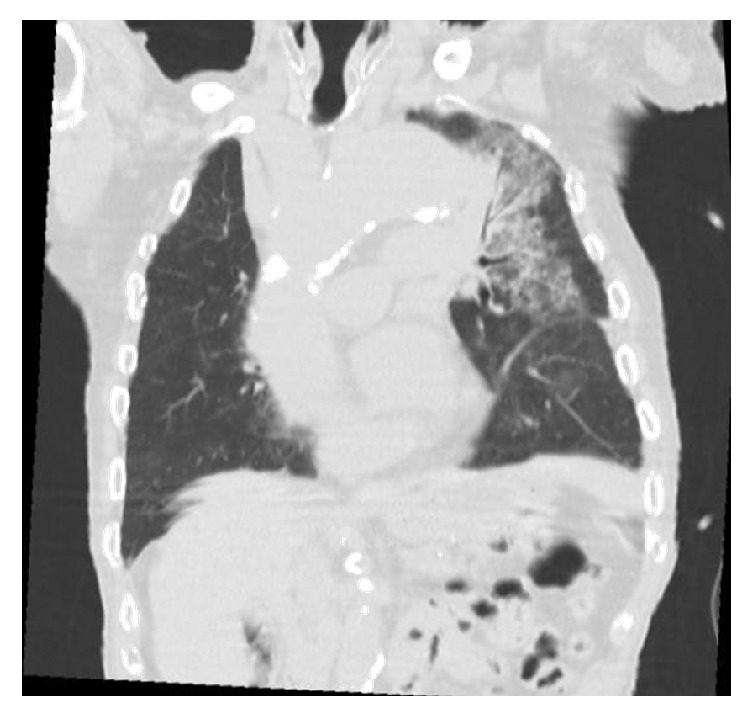
Coronal view aortic aneurysm eroding into the left upper lobe.

A ninety-year-old man with no significant medical history presented to the emergency department with abrupt onset of hemoptysis. The patient was hemodynamically stable. Lung exam revealed bilateral lung crackles. His chest X-ray showed left upper lobe opacity with widened mediastinum due to aortic dilatation (Figure 1). CT of the chest revealed the aneurysmatic dilatation of the ascending arch and the descending aorta and pulmonary infiltrates in left upper lobe suspicious of aorto-bronchial fistula with bleeding in the lung (Figures 2, 3, and 4). The patient was started on supportive care, including intubation and mechanical ventilation. Cardiology and Cardiothoracic Surgery were consulted. Cardiothoracic surgery agreed with the diagnosis of ascending aortic aneurysm and bronchopulmonary fistula; however due to his poor functional status and overall frailty the risk of surgery outweighed the benefits; hence surgical intervention was not pursued. During his second day of hospitalization the patient developed another episode of massive hemoptysis resulting in hypovolemic shock, and despite the best efforts of the medical personnel, the patient expired.

## 2. Background

Aortic aneurysm is a permanent localized arterial dilation more than 50 % of the average diameter of the aorta [1]. Among the complications of an aortic aneurysm one of the rarest is the aortobronchial fistula which presents with massive hemoptysis; this condition is lethal if not treated surgically. Diagnostic workup consists of CT angiography, echocardiography, and MRI. The mainstay treatment is immediate surgical repair [2].

## 3. Discussion

The mechanism of the injury in the fistula is related to the expansion of an aortic aneurysm, which causes compression in the tracheobronchial tree, which triggers a chronic inflammatory response. Rupture may be confined to the tracheobronchial tree or may extend into the pleural space [3]. If ABF is manifested as massive hemoptysis, early diagnosis is vital given its high mortality if untreated. The gold standard for diagnosis is CT angiography [4–6].

Preoperative management of the hemodynamically unstable patient consists of endotracheal intubation with a single lumen or double lumen endotracheal tube, initiation of invasive mechanical ventilation, and blood transfusions [4]. The definite treatment will be surgical correction. The surgical technique depends on the type of aneurysm, the location of the fistula, the patient medical history, and the hemodynamic state [6].

Open surgery along with cardiopulmonary bypass technique consists in thoracotomy, with total aortic root replacement with composite graft, and prosthetic aortic valve replacement with replantation of the coronary arteries. The fistula is repaired by separating and ligating the communications with silk [3]. This technique used to be the first therapeutic option for the last 40 years but it had perioperative mortality of 15-41% [5–8]. Thoracic Endovascular Aneurysm Repair (TEVAR) a less invasive procedure, which started as an alternative therapeutic option and has now become the treatment of choice for ABF with lower mortality rate (5.9%) [8]. The procedure consists of introducing a stent via the femoral artery and through angiography the stent is placed in the desired landing zone and the graft is left in the desired region. Although TEVAR has become the standard treatment, the technique does not address the defect in the respiratory tract [4–9]. Potential risks include a recurrence of the ABF and infection of the stent graft, though TEVAR showed lower morbidity mortality rate and good postoperative outcome in the short and mid-term [10]. A third alternative is the combination of these procedures with TEVAR first for the vascular repair and once the patient's condition improves a thoracotomy is done for the respiratory tract repair [9].

## 4. Conclusion

Although aortobronchial pulmonary fistula is an extremely rare cause of hemoptysis, it must still be kept as part of the differential diagnosis. The management of this condition is mainly surgical, the alternative being TEVAR. Our primary recommendation is to contact cardiothoracic surgery as soon as the diagnosis is suspected.
